# A Quantitative Real-Time PCR-Based Strategy for Molecular Evaluation of Nicotine Conversion in Burley Tobacco

**DOI:** 10.3390/ijms161126038

**Published:** 2015-11-17

**Authors:** Bo Sun, Sheng-Ling Xue, Fen Zhang, Zhao-Peng Luo, Ming-Zhu Wu, Qing Chen, Hao-Ru Tang, Fu-Cheng Lin, Jun Yang

**Affiliations:** 1 College of Horticulture, Sichuan Agricultural University, Chengdu 611130, China; sunadam011@163.com (B.S.); 18161250603@163.com (S.-L.X.); zhangf_12@163.com (F.Z.); supnovel@gmail.com (Q.C.); 2 National Tobacco Gene Center, Zhengzhou Tobacco Research Institute, Zhengzhou 450001, China; luozhaopeng163@163.com (Z.-P.L.); mingzhuwus@126.com (M.-Z.W.); fuchenglin@zju.edu.cn (F.-C.L.)

**Keywords:** *CYP82E4*, burley tobacco, nicotine conversion, quantitative real-time PCR (qPCR)

## Abstract

Nornicotine production in *Nicotiana tabacum* is undesirable because it is the precursor of the carcinogen *N*′-nitrosonornicotine. In some individual burley tobacco plants, a large proportion of the nicotine can be converted to nornicotine, and this process of nicotine conversion is mediated primarily by enzymatic *N*-demethylation of nicotine which is controlled mainly by *CYP82E4*. Here we report a novel strategy based on quantitative real-time polymerase chain reaction (qPCR) method, which analyzed the ratio of nicotine conversion through examining the transcript level of *CYP82E4* in burley leaves and do not need ethylene induction before detected. The assay was linear in a range from 1 × 10^1^ to 1 × 10^5^ copies/mL of serially diluted standards, and also showed high specificity and reproducibility (93%–99%). To assess its applicability, 55 plants of burley cultivar Ky8959 at leaf maturing stage were analyzed, and the results were in accordance with those from gas chromatograph-mass spectrometry (GC-MS) method. Moreover, a linear correlation existed between conversion level and *CYP82E4* transcript abundance. Taken together, the quantitative real-time PCR assay is standardized, rapid and reproducible for estimation of nicotine conversion level *in vivo*, which is expected to shed new light on monitoring of burley tobacco converter.

## 1. Introduction

In most commercial varieties of cultivated tobacco (*Nicotiana tabacum* L.), the predominant alkaloids are nicotine and nornicotine, accounting 94%–97% and 2%–5% of total alkaloid content, respectively [1,2,3]. These plants are frequently referred to as “nonconverters”. In some of the others, however, a large percentage (as high as 95%) of nicotine is metabolized to nornicotine under the catalysis of the nicotine *N*-demethylase. Hence the corresponding plants are termed “converters”. This above enzymatic metabolism of nicotine to nornicotine is termed as “conversion” [3,4]. Generally, the frequency of nicotine conversion in burley tobacco is notably higher than that in flue-cured tobacco [2,3].

The accumulation of nornicotine instead of nicotine in *N. tabacum* is undesirable. Nornicotine is the precursor of *N*’-nitrosonornicotine (NNN), a tobacco-specific nitrosamines (TSNA), which has been proven to be a strong carcinogen in lab animals [5,6,7]. In addition, a high content of nornicotine was also indicated to decline tobacco quality via producing smoking characteristics and undesirable flavor [8,9]. Therefore, how to find and remove the converters in burley tobacco population is crucial for tobacco production and quality control. Low conversion (LC) Protocol is a common method on monitoring the nicotine conversion in burley tobacco population [10]. Based on this LC Protocol, nowadays the most common detection procedures are potential induction first to maximize conversion through ethylene (ETH) treatment, and then content measurement through gas chromatograph-mass spectrometry (GC-MS) and/or gas chromatograph (GC) [11,12]. However, these approaches required a lot of sampling, some pre-treatments after leaf harvest and time-consuming. This report described the development of a novel molecular assay that can estimate the level of nicotine conversion in burley tobacco leaves at specific stage. We first prepared the external standards and chose the specific primers. Then, the nicotine conversion content was evaluated by quantitative real-time polymerase chain reaction (qPCR) method. To assess its practicability, 55 plants of burley cultivar Ky8959 at leaf maturing stage were used. We compared the data with those obtained by GC-MS method, and the correlation was also analyzed.

## 2. Results

### 2.1. Development of Quantitative Real-Time PCR Assay

#### 2.1.1. Gel Electrophoresis

**Figure 1:**
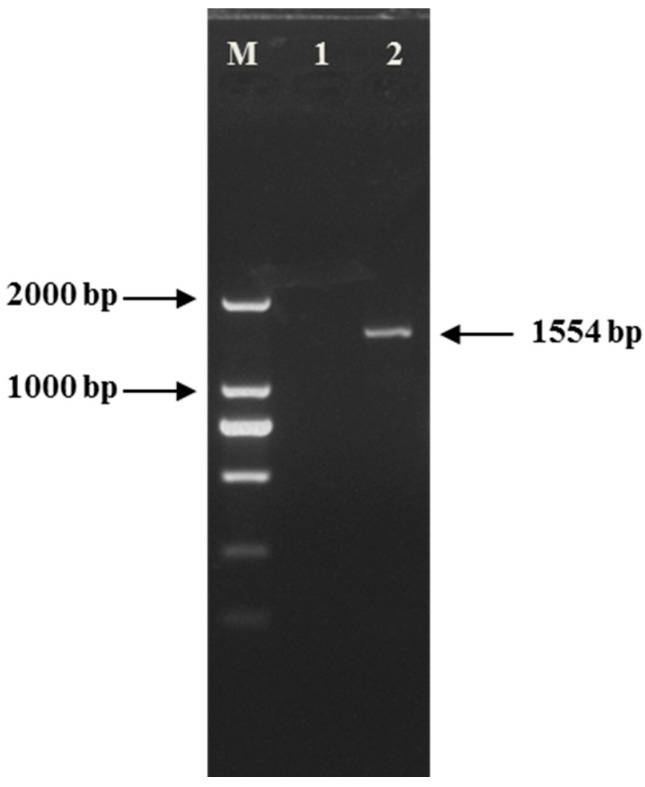
Agarose gel electrophoresis of *CYP82E4* cDNA displaying a single, specific band at 1554 bp. cDNA was isolated and amplified via a conventional PCR with *CYP82E4-cDNA* primers. M: Takara DL2000 DNA Marker; 1: negative control (double-distilled H_2_O); 2: PCR product of *CYP82E4* cDNA.

*CYP82E4* cDNA was successfully amplified with the Thermocycler instrument. The isolated cDNA from leaves indicated a single band by gel electrophoresis, which represented the specific amplicon (Figure 1). The same DNA sequence of 10 randomly selected clones displayed that the amplicons owned with a single cDNA sequence. The cDNA contained 1554 nucleotide base pairs, which encoded a protein of 518 amino acid residues. BLAST homology searches showed that the isolated cDNA sequence shared 99.9% identity with *Nicotiana tabacum CYP82E4v2* (GenBank number: DQ131885.1). Because of the high sequence similarity to *CYP82E4v2*, the isolated cDNA was confirmed as *CYP82E4*.

#### 2.1.2. Sensitivity of Quantitative Real-Time PCR Assay

**Figure 2:**
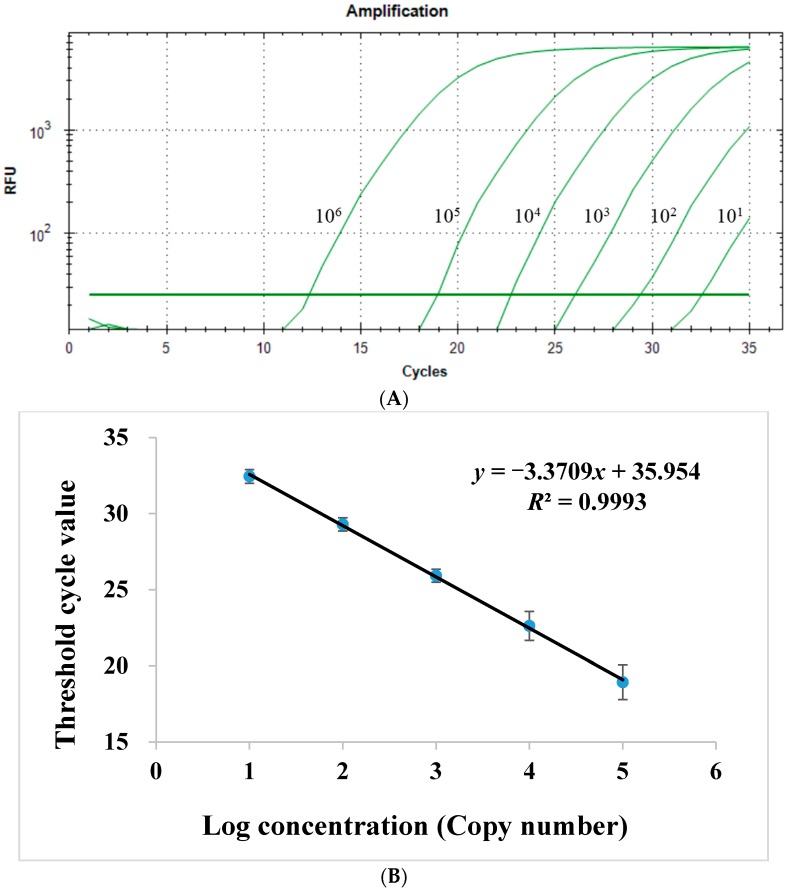
Quantification (**A**) and the standard curve (**B**) of serially diluted standards of *CYP82E4* recombinant vectors by the quantitative real-time PCR technique.

**Figure 3:**
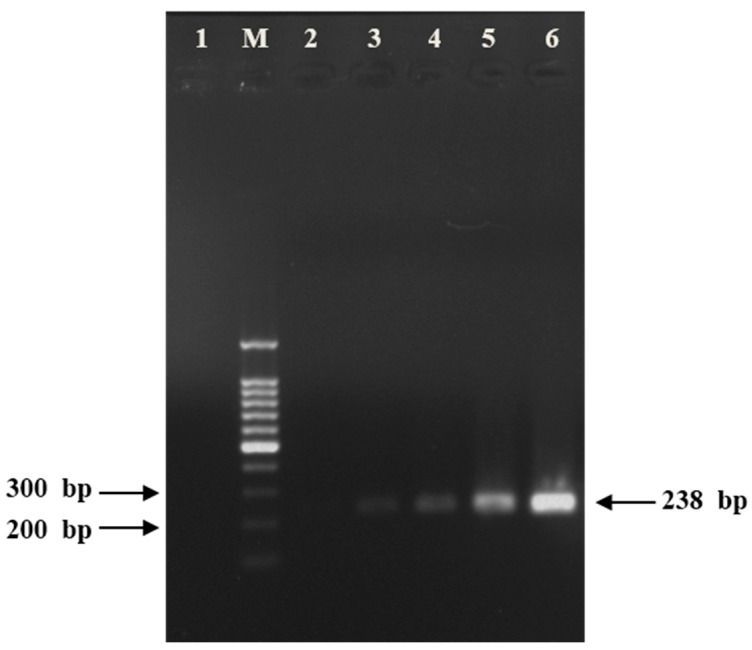
Agarose gel electrophoresis of the serially diluted standards showing a single, specific band at 238 bp. cDNA was isolated and amplified via a conventional PCR with *CYP82E4-RT* primers (35 cycles). M: Takara 100 bp DNA ladder marker; 1: negative control (double-distilled H_2_O); 2: 1 × 10^2^ copies/mL; 3: 1 × 10^3^ copies/mL; 4: 1 × 10^4^ copies/mL; 5: 1 × 10^5^ copies/mL; 6: 1 × 10^6^ copies/mL.

For sensitivity testing, serially diluted recombinant vectors were examined by the qPCR technique described in Experimental Section, and the results demonstrated a sensitivity of 1 × 10^1^ copies/mL for *CYP82E4* transcript level (Figure 2A). The results of conventional PCR displayed that the band of 1 × 10^2^ copies/mL vector could not be detected, which implied the high sensitivity of the qPCR method, and proved the sensitivity level at the same time (Figure 3).

#### 2.1.3. Specificity and Reproducibility of Quantitative Real-Time PCR Assay

**Figure 4:**
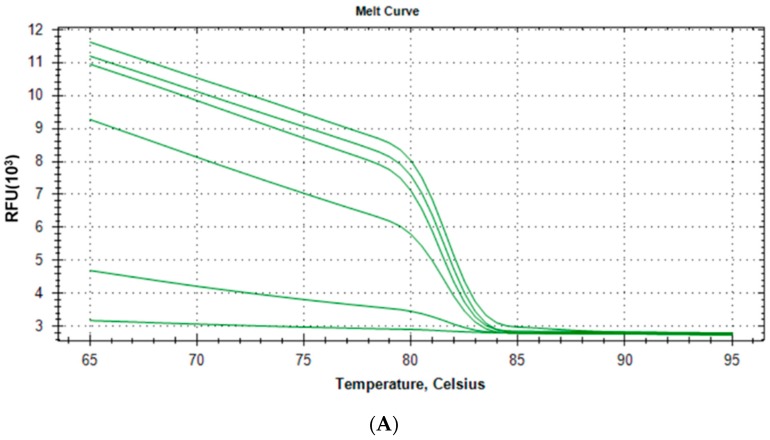

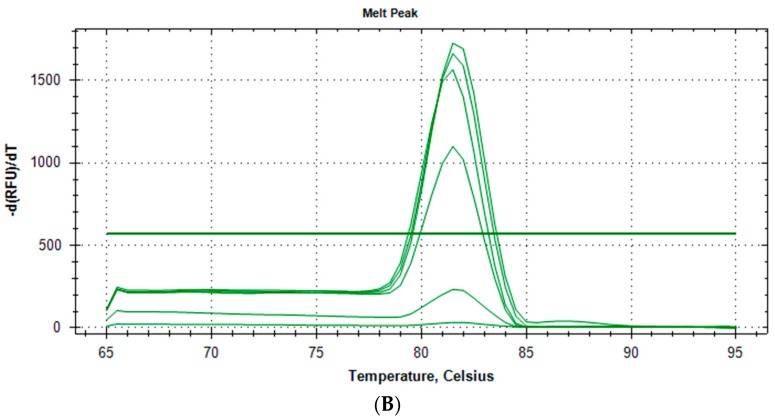
Melt curve (**A**) and melt peak (**B**) of serially diluted standards by the quantitative real-time PCR technique.

Melt curve and melt peak were examined for specificity of qPCR assay (Figure 4). The melt peaks were all consistent and the temperature was 82 °C. Amplification of serially diluted standards was repeated five times from the same recombinant vector (Table 1). The coefficient of variation within batches obtained of serial dilution of standards (1 × 10^1^–1 × 10^5^ copies/mL) by qPCR assay ranged from 1.39% to 6.49%. In order to get rid of variations during the extraction procedure, the qPCR assay was repeated five times of different serial dilutions of recombinant vectors. The results showed that all the coefficient of variation between batches was less than 10%, and the highest value was merely 7.12% (1 × 10^5^ copies/mL), which displayed that the sensitivity was also reproducible (Table 1).

**Table 1:** The reproducibility of quantitative real-time PCR method for molecular detection of nicotine conversion.

The Standard Concentration (copies/mL)	Variation Coefficient within Batches (%)	Variation Coefficient between Batches (%)
1 × 10^1^	1.39	2.71
1 × 10^2^	1.47	2.46
1 × 10^3^	1.65	2.83
1 × 10^4^	4.21	4.72
1 × 10^5^	6.49	7.12

#### 2.1.4. Linear Range of Quantitative Real-Time PCR Assay

The linear range of qPCR assay was from 1 × 10^1^ to 1 × 10^5^ copies/mL of standard. Figure 2B plots the results of standard curve for serially diluted *CYP82E4* recombinant vectors.

### 2.2. Application of Quantitative Real-Time PCR Assay with Test Samples

Fifty-five plants collected from burley cultivar Ky8959 at leaf maturing stage (Growth stage 43–47, [13]) were used to verify the effect of the qPCR method. After total RNAs were isolated, the RNA concentration of each sample was measured, and diluted to 100 μg/mL. This step could warrant the same efficiency obtained among various samples.

**Figure 5:**
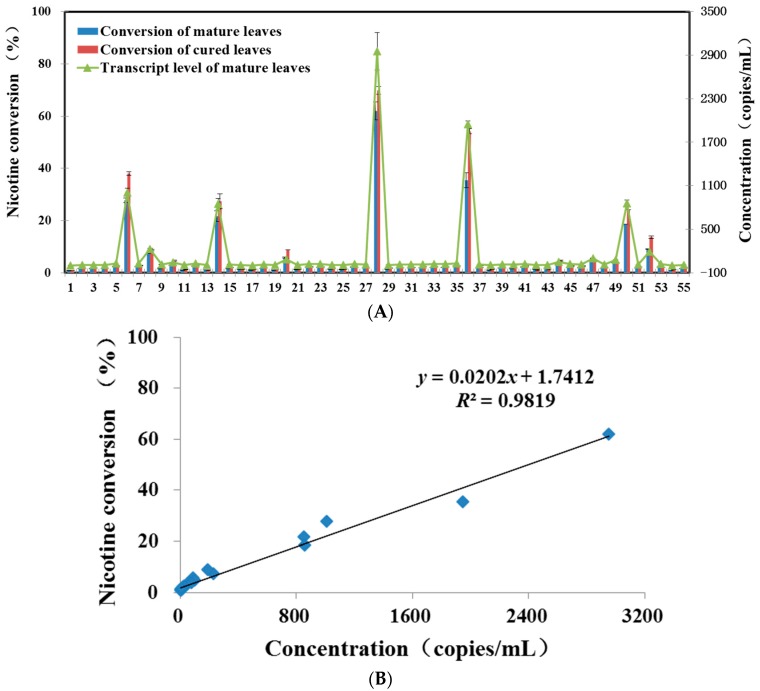
The relationship of nicotine conversion ratios and *CYP82E4* transcript levels in Ky8959 plants. (**A**) The nicotine conversion ratios and transcript levels of Ky8959 plants. Fifty-five plants were used as materials. The nicotine conversions of matured leaves (Growth stage 43–47, [13]) and cured leaves were analyzed by GC-MS method, while the transcript levels of matured leaves were detected by qPCR method. Each biological sample was analyzed with two technical replicas; (**B**) Linear correlation (*R*^2^ = 0.9819) between the nicotine conversion and corresponding transcript levels in matured leaves. The blue boxes represented the nicotine conversion ratios and *CYP82E4* transcript levels of Ky8959 plants. Each biological sample was analyzed with two technical replicas.

The *CYP82E4* transcript levels of Ky8959 plants were detected using the qPCR method. The results indicated that the difference of concentration among samples was relatively large. The top value (2952 copies/mL) was found in No. 28, which was 1114 times higher than the lowest level (2.65 copies/mL) in No. 54 (Figure 5A).

### 2.3. Compared with GC-MS Method

The nicotine conversions of test samples were also analyzed by GC-MS method to confirm the accuracy of the molecular evaluation method. In order to understand the change during the curing process, the conversion ratios of corresponding Ky8959 plants were also analyzed by GC-MS. The conversion of No. 36 exhibited the biggest change and the value increased by 1.53-fold (Figure 5A). On the contrary, conversion ratios of six plants slightly decreased after curing. On the whole, the conversion ratios of most plants enhanced slightly in curing samples in comparison with mature leaves. GC-MS analysis indicated that they contained the same trends with the qPCR assay. The nicotine conversions of No. 28 mature leaf topped in plants population, and the ratio was as high as 62%, while the lowest ratios was found in No. 1 and No. 54, which were both less than 1%, This result was also in accordance with that of qPCR transcript assay (Figure 5A). The linear relationship between nicotine conversion level and *CYP82E4* transcript abundance was also investigated (Figure 5B). As shown here, it indicated a significantly positive correlation and a value of *R*^2^ of 0.9819.

## 3. Discussion

qPCR has been used successfully to detect transcript levels that are difficult to accurately quantitate by normal PCR method [14]. In the current study, we analyzed the sensitivity, specificity and reproducibility of qPCR assay and the possibility to apply this method to monitor the nicotine conversion level in burley samples. To estimate the correctness of the qPCR assay, 55 Ky8959 plants at leaf maturing stage were analyzed via both this assay and GC-MS method, the results obtained by qPCR assay were consistent with those of GC-MS method, and all samples values tested qPCR assay were within the linear range.

This qPCR method has shown some obvious differences compared with the GC-MS analytical method. Firstly, the sample is *in vivo* and the quantity needed by qPCR assay was remarkably decreased as low as 0.1 g rather than GC/GC-MS approach, while barely affects plant growth and non-damage. Secondly, the qPCR assay did not need some pre-treatments, which could notably simplify the detection process and save analytical time. The procedure of nicotine conversion detection in previous studies was generally as follows: individual leaves were excised, and treated by ethephon or sodium bicarbonate because they could promote the demethylation of nicotine to nornicotine. The treated tobacco leaves were put in small plastic storage bags at room temperature in the dark until they turned yellow. The alkaloid analysis was carried out by GC or GC-MS determination, and the nicotine conversion was subsequently calculated [3,10,11]. Instead, the result of qPCR assay could be obtained in a few hours after sampling.

Compared to flue-cured tobaccos, burley tobaccos generally are unstable for conversion, and ETH treatment is regularly used to maximize conversion before GC detection [3,10]. Thus, the potential is very important to the conversion issue. However, in the present study we are trying to find a suitable point, which does not need additional induction. Our result suggested that leaf maturing stage is the key point to achieve the goal without ETH treatment. It could simplify the process to a certain extent.

Moreover, absolute quantification was performed in qPCR assay, and the absolute transcript copy number could be calculated in our study. In contrast with relative quantification, this method overcame the obstacles and offered more comparability and accuracy for samples analysis with different batches, reagents, reference genes, instrument models and so on. Meanwhile, the RNA extraction method used for detection is crucial, because the total amount of RNA may vary greatly from sample to sample. Therefore, good quality reagents should be adopted for total RNA extraction, and the RNA concentration should be measured routinely for all samples by spectrophotometer, with diluting samples to the same concentration before qPCR analysis.

The accumulation of plant secondary metabolite is largely determined by the related transcript levels [15]. The key to success of qPCR assay for estimation of nicotine conversion in our study is the close relationship between *CYP82E4* transcript level and nicotine conversion ratio. The cultivated tobacco (*N. tabacum*) is an allotetraploid species came from the hybridization of ancestral *N. sylvestris* and *N. tomentosiformis* [16]. Different from *N. tabacum*, *N. sylvestris* and *N. tomentosiformis* convert nicotine to nornicotine in the green and senescing leaves, respectively [17]. Previous research demonstrated the molecular basis of nicotine *N*-demethylation showed that a cytochrome P450 gene, designated *NtabCYP82E4*, catalyzed the *N*-demethylation of nicotine in yeast and *N. tabacum*, and the gene expression of *NtabCYP82E4* was mightily induced in the senescing leaves of converter tobacco [18,19]. Moreover, the expression of *NtabCYP82E4* RNAi mutant indicated effectively lowered the nornicotine level in senescing leaves of high converter plants [3,9], and genetic analyses indicated that the unstable mutation occured at the *NtabCYP82E4* locus [17,20]. Afterwards, other nicotine *N*-demethylase genes (*CYP82E2*, *CYP82E3*, *CYP82E5*, and *CYP82E10*) were successively isolated form *Nicotiana* species [12,17,19,20]. However, *NtabCYP82E2* and *NtabCYP82E3* in nonconverter plants were impaired through the substitutions of amino acid, and did not encode nicotine *N*-demethylase (NND) [3,17,20]. Moreover, *CYP82E5* and *CYP82E10* had mainly played the role on nicotine conversion in root, and were considered as “minor” nicotine *N*-demethylase genes in leaf [12,19]. Therefore, those findings demonstrated that *CYP82E4* plays a main role in mediating nicotine *N*-demethylation in *N. tabacum* leaf, and provided a possibility to reflect the nicotine conversion through the detection of *CYP82E4* transcript level. Our results showed that a linear correlation (*R*^2^ = 0.9819) existed between conversion level and *CYP82E4* transcript abundance (Figure 5B). Therefore, in our study, the cDNA of *CYP82E4* was isolated for preparing the external standards and the transcript level could be used to evaluate the nicotine conversion of samples. The result has perfectly proven the importance of *CYP82E4* to nicotine conversion. However, the enzymatic activity does not always follow transcript levels. Therefore, we will still examine the correctness via more burley cultivars and populations in the future.

In this study, qPCR method was established to detect the level of nicotine conversion. On the other hand, how to reduce the levels of nicotine conversion is also the focus of scientific research. Previous studies reported that single or all three *NND* genes were mutated by RNAi or EMS treatment, and the nornicotine levels in mutant plants were much lower than that found in conventional burley tobacco [9,11,12]. Therefore, it is indicated that the next accepted technology for dealing with the problem of nicotine conversion is mutations in the three known *NND* genes.

## 4. Experimental Section

### 4.1. Development of Quantitative Real-Time PCR Method

#### 4.1.1. Preparation of the Standard of Quantitative Real-Time PCR

Matured leaf samples (5 g) for preparation of the standard were collected from burley tobacco cultivar “B37” planted in the greenhouse of Zhengzhou Tobacco Research Institute (Zhengzhou, China). For isolation of total ribonucleic acid (RNA), leaf samples were ground and homogenized in liquid nitrogen. Total RNA was isolated using RNeasy Plant Mini kit (Qiagen, Hilden, Nordrhein-Westfalen, Germany) according to the manufacturer’s instructions. The RNA concentration and quality were assessed through photometric measurement (GE, Piscataway, NJ, USA) and gel electrophoresis. Approximately 2 μg total RNA was utilized to synthesize single stranded complementary deoxyribonucleic acid (cDNA) with AMV reverse transcriptase (Takara, Japan) and oligo-dT 18 primers, as described by manufacturer’s instructions.

The cDNA was used as the template, and PCR of *CYP82E4* amplification was performed in a total volume of 50-μL containing 1 μL of cDNA, 1 μL each primer (20 μM), 8 μL dNTP mixture (2.5 mM each), 5 μL PCR Buffer II (Mg^2+^ Plus) and 0.5 μL LA Taq (5 U/μL) (Takara, Tokyo, Japan). The nucleotide sequences of the specific primers (*CYP82E4-cDNA*) (forward, 5′-ATGCTTTCTCCCATAGAAGCC-3′; reverse, 5′-TTAATAAAGCTCAGGTGCCAG-3′) were used in the PCR as previously described [20]. The PCR program was carried out at 95 °C for 30 s, followed by 55 °C for 30 s and 72 °C for 90 s for 30 cycles. PCR products were visualized on a 1% ethidium bromide-stained agarose gel. The predominant fragment of 1554 bp was purified after being excised from the gel, and cloned into the pMD19-T vector (Takara, Japan), and then sequenced at Sangon Biotech (Shanghai) Co., Ltd. (Shanghai, China). The recombinant vector was stored at −20 °C and used as the standard of detecting the level of nicotine conversion.

#### 4.1.2. Quantification of the Standard

For quantification of the external standards, 3 μL aspiration of purified recombinant vector was used to measure the concentration and OD_260_/OD_280_ by NanoVue Plus spectrophotometer (GE, Piscataway, NJ, USA). The copy number was calculated subsequently. The mother liquor of purified recombinant vector was diluted serially by sterile water, and the concentration of dilution solution was from 1 × 10^6^ to 1 × 10^1^ copies/mL.

#### 4.1.3. Quantitative Real-Time PCR Analysis

Various concentrations of *CYP82E4* standards were amplified using a Bio-Rad iCycler thermocycler (Bio-Rad, Hercules, CA, USA) in a total reaction volume of 20 μL, which contains 10 μL 2 × SYBR Premix EX Taq (Takara, Japan), 2 μL of diluted recombinant vector and 1 μL of each gene-specific primer (10 μM). The nucleotide sequences of primers (*CYP82E4-RT*) (forward, 5′-ACGTGATCCTAAACTCTGGTCTG-3′; reverse, 5′-GCCTGCACCTTCCTTCATG-3′) were used as previously described [20]. The qPCR was performed according to the following protocol: 95 °C for 2 min; 35 cycles of 95 °C for 30 s, 55 °C for 30 s, 72 °C for 50 s followed by final extension at 72 °C for 5 min [20]. The specificity of the reactions was confirmed by qPCR standard melt curve method. The data were expressed as threshold cycle value. Each assay was repeated three times. The results of various concentrations of standards were utilized to make the standard curve.

#### 4.1.4. Gel Electrophoresis

After each Light Cycler run, agarose gel electrophoresis was conducted to have an independent validation check of the presence of an amplicon. To detect the length of the amplicon generated, DL2000 DNA marker and 100 bp DNA ladder marker (Takara, Japan) were used.

### 4.2. Evaluation of Nicotine Conversion by Quantitative Real-Time PCR Method

The qPCR assay was used to analyze burley cultivar Ky8959. The tobacco plants were grown on the experimental station of Zhengzhou Tobacco Research Institute at Enshi Autonomous Prefecture, Hubei Province, China, which is the best and largest area for burley tobacco production in China. Fifty-five plants were randomly selected, numbered and sampled both at leaf maturing stage (Growth stage 43–47 [13]) and leaf curing stage (30 days after harvesting). Ten leaf discs with 0.5 cm in diameter were punched in each leaf, and then mixed for RNA extraction. The positions of the leaf discs were 3A, 6A, 8A, 2B, 5B, 7B, 9B, 4C, 8C and 6D, which according with Burton (1992) protocol [2]. Total RNAs of matured leaves were isolated according to the protocol above. Whereafter, the RNA concentration of each sample was measured, and then diluted to 100 μg/mL, respectively. The single stranded cDNAs of test samples were synthesized and diluted 10 times for qPCR analysis. Meanwhile, parts of the samples were lyophilized with freeze dryer (VirTis Inc., New York, NY, USA). The dry materials were ground into a fine powder using a coffee mill and stored at −20 °C for alkaloid analysis.

### 4.3. Analysis of Nicotine Conversion by GC-MS Method

Alkaloids were extracted from lyophilized samples and analyzed as previously described with a minor modification [21,22]. A 300 mg sample was added to 2.0 mL of 5% NaOH in a 50 mL conical flask until mixture, and then incubated for 15 min at room temperature. Alkaloids were extracted by addition of 20 mL extraction solution (0.01% triethylamine (Fluka, St. Louis, MO, USA)/chloroform (Merck, Darmstadt, Hesse-Darmstadt, Germany)) and ultrasonic extracted for 15 min at 20 °C. Following phase separation, an aliquot of the organic phase was filtered through a column filling with anhydrous sodium sulfate. Two milliliters of the filtrate was transferred to a sample vial and subjected to GC-MS analyses.

Qualitative and quantitative analyses of alkaloids in burley tobacco were conducted using an Agilent 7890A gas chromatograph (GC) interfaced to an Agilent 5975C mass-selective detector (Agilent, Santa Clara, CA, USA), which was controlled by a Agilent G1701EA GC-MSD ChemStation. The GC was equipped with a HP-35 capillary column (Agilent), 30 m × 0.250 mm, and 0.25 μm film thickness. Helium was used as a carrier gas with a flow of 1.0 mL·min^−1^. The temperature of the oven was set initially at 100 °C for 3 min, and programmed at 8 °C·min^−1^ to 260 °C and finally held for 10 min. Injector temperature was 250 °C set at split mode (10:1) with an injection volume of 2 μL. The mass detector was operated by electron impact ionization (EI, 70 eV) with an ion source temperature of 230 °C and an interface temperature of 280 °C. The mass spectrometer was operated in selected ion monitoring (SIM) mode for quantitative analysis [21,22]. The alkaloid content was determined through a calibration curve, and the percentage of nicotine conversion was calculated as: [% nornicotine/(% nornicotine + % nicotine)] × 100 [11].

### 4.4. Statistical Analysis

Statistical analysis was performed via the Statistical Product and Service Solutions (SPSS) package program version 11.5 (SPSS Inc. Chicago, IL, USA). Data were analyzed by one-way ANOVA model, followed by the Least Significant Differences (LSD) test at a 95% confidence level (*p* < 0.05). The values were represented as means with standard error.

## 5. Conclusions

In conclusion, quantitative real-time PCR assay described in this study allowed sensitive and specific estimation of the nicotine conversion levels in burley samples *in vivo* at specific stage. The results from qPCR assay were consistent with those from GC-MS method, which meant the qPCR assay is of great application value. Therefore, the qPCR assay is a valuable and promising tool for monitoring of nicotine conversion in burley samples.
